# Time-dependent prediction of arrhythmia recurrences during long-term follow-up in patients undergoing catheter ablation of atrial fibrillation: The Leipzig Heart Center AF Ablation Registry

**DOI:** 10.1038/s41598-019-43644-2

**Published:** 2019-05-08

**Authors:** Jelena Kornej, Katja Schumacher, Samira Zeynalova, Philipp Sommer, Arash Arya, Manuela Weiß, Christopher Piorkowski, Daniela Husser, Andreas Bollmann, Gregory Y. H. Lip, Gerhard Hindricks

**Affiliations:** 1 Department of Electrophysiology, Heart Center Leipzig at University of Leipzig, Leipzig, Germany; 2 Institute for Medical Informatics, Statistics, and Epidemiology, University of Leipzig, Leipzig, Germany; 3 Clinic of Electrophysiology, Heart and Diabetes Center NRW, University Hospital of Ruhr- University Bochum, Bad Oeynhausen, Germany; 4 Department of Electrophysiology, Heart Center, University of Technology Dresden, Dresden, Germany; 5 Liverpool Centre for Cardiovascular Science, University of Liverpool and Liverpool Heart & Chest Hospital, Liverpool, United Kingdom; 6 Aalborg Thrombosis Research Unit, Department of Clinical Medicine, Aalborg University, Aalborg, Denmark

**Keywords:** Outcomes research, Atrial fibrillation

## Abstract

The prediction of arrhythmia recurrences after catheter ablation of atrial fibrillation (AF) remains challenging. The aim of current analysis was to investigate the time-dependent prediction of arrhythmia recurrences after AF catheter ablation during long-term follow-up. The study included 879 patients (61 ± 10 years; 64% males; 39% persistent AF) undergoing first AF catheter ablation. Rhythm outcomes were documented using 7-days Holter monitoring. The APPLE score (Age, Persistent AF, imPaired eGFR, Left atrium (LA), EF) was calculated at baseline, while MB-LATER score (Male gender, Bundle branch block, LA, AF Type, Early Recurrences) 3 months after ablation. The median follow-up time was 37 months [95%CI 35;39]. ERAF and LRAF occurred in 45% and 64%, respectively. On multivariable analysis, ERAF (HR 2.095, 95%CI 1.762–2.490, p < 0.001) was strongly associated with LRAF. The APPLE (HR 1.385, 95%CI 1.276–1.505, p < 0.001) and MB-LATER (HR 1.326, 95%CI 1.239–1.419, p < 0.001) scores significantly predicted LRAF during follow-up. On the ROC analysis, APPLE (AUC 0.640, 95%CI 0.602–0.677, p < 0.001) and MB-LATER (AUC 0.654, 95%CI 0.616–0.691, p < 0.001) demonstrated moderate prediction. Summarizing, ERAF was the strongest predictor for LRAF in time-dependent manner. The APPLE and MB-LATER scores demonstrated moderate prediction of arrhythmia recurrences during long term follow-up.

## Introduction

Catheter ablation targeting the pulmonary veins still remains the most important therapeutic strategy in atrial fibrillation (AF) treatment with continuous escalation of its popularity since the late 1990s^1,2^. In most patients catheter ablation is superior to antiarrhythmic drugs, however, up to 30–50% of ablated AF patients suffer recurrences within the first year^3^. AF recurrences are associated with impaired quality of life and relate to increased morbidity and mortality because of relevant cardio- and cerebrovascular events^1,4^. Also, recurrences lead to repeated ablation procedures and higher hospitalization rates and treatment costs^5^. All this explains a considerable clinical interest to predict the risk for recurrences already before invasive procedure with the goal to shape personalized strategies in AF patients.

There have been multiple studies analysing the impact of different scores on recurrence prediction – from the CHADS_2_ and CHA_2_DS_2_-VASc scores (designed for the thromboembolic risk prediction) to the specific rhythm outcomes prediction scores as ALARMEC, BASE-AF2, CAAP-AF, ATLAS and LAGO^6–13^.

The APPLE score was originally developed to predict AF recurrences within the first year after catheter ablation^14^. In contrast, the MB-LATER score has been introduced to predict very late arrhythmia recurrences in arrhythmia-free patients within first 12 months after ablation^15^. Recently, the predictive ability of APPLE and MB-LATER scores had been proven to predict electro-anatomical substrate indicating advanced disease stage and explaining poor ablation success in such patients^16^. However, the time-dependent prediction of arrhythmia recurrences during very long follow-up period using clinical variables, in addition to APPLE and MB-LATER scores have not been studied and was the aim of current analysis.

## Methods

### Study population

The study population consisted of 879 patients from The Leipzig Heart Center AF Ablation Registry, which included consecutive high-symptomatic patients presenting for the catheter ablation between January 2007 and December 2011^14^. The study was approved by the local Ethical Committee (Medical Faculty, University Leipzig) and performed according to the Declaration of Helsinki and Institutional Guidelines. All patients provided written informed consent for participation.

Paroxysmal and persistent AF was defined according to current guidelines^5^. Paroxysmal AF was defined as self-terminating within first 48 hours and up to 7 days after onset documented by previous routine electrocardiograms (ECG) or Holter ECG. Persistent AF was defined as any AF episode either lasting longer than 7 days or requiring drug or direct current cardioversion for termination. In all patients, transthoracic and transesophageal echocardiography was performed prior to ablation. All class I or III antiarrhythmic medications with the exception of amiodarone were discontinued at least 5 half-lives before the procedure.

The APPLE (one point for Age >65 years, Persistent AF, imPaired eGFR <60 ml/min/1.73 m^2^, Left atrial (LA) diameter ≥43 mm, EF < 50%) was calculated before catheter ablation using baseline patients’ characteristics, while the MB-LATER scores (one point for Male gender, Bundle branch block or QRS >120 ms, LA diameter ≥47 mm, AF Type (persistent AF), Early Recurrence <3 months) was calculated 3 months after ablation, when the data regarding early recurrences were available^15,16^.

### Catheter ablation

AF catheter ablation was performed using a well-documented approach as previously described^14^. Briefly, patients presenting with AF at the beginning of the procedure were electrically cardioverted and ablation was performed during sinus rhythm (SR) (i.e., AF termination with ablation was not attempted). In all patients, circumferential LA ablation lines were placed around the antrum of the ipsilateral pulmonary veins (irrigated tip catheter, pre-selected tip temperature of 48 °C, and maximum power of 30–50 W). In patients with persistent AF, additional linear lesions were added at the LA roof, the basal posterior wall and the LA (mitral) isthmus. At the end of procedure, linear block was confirmed across the roof and the mitral isthmus. After circumferential line placement, voltage and pace mapping along the ablation lines were used to identify and close gaps. The isolation of all pulmonary veins with bidirectional block was verified with a multipolar circular mapping catheter and was defined as the procedural endpoint.

After ablation, class I and III antiarrhythmic drugs were routinely not reinitiated. Proton pump inhibitors were added for 4 weeks. According to the current guidelines, oral anticoagulation was prescribed for 3–6 months after catheter ablation and continued subsequently depending on stroke risk stratification using the CHA_2_DS_2_-VASc score thereafter^5^.

### Follow-up

All patients were followed in the outpatient clinic after catheter ablation. During the follow-up period, 7-days Holter ECG recordings were performed (immediately, 3, 6 and 12 months after the ablation, then every 6 months). Additional resting ECGs and Holter ECG recordings were obtained when patients’ symptoms were suggestive of AF. Arrhythmia recurrences were defined as early (ERAF, occurring within 3 months after ablation) and late (LRAF, over 3 months period). If electrical or pharmacologic cardioversion and/or repeat procedure were needed, this was also considered as arrhythmia recurrence, i.e. a study endpoint.

### Statistical analysis

Data are presented as the mean/standard deviation for normally distributed or median/interquartile range (IQR) for skewed continuous variables and as proportions for categorical variables. Continuous variables were tested for normal distribution using the Kolmogorov–Smirnov test. The differences between continuous values were assessed using an unpaired two-tailed t-test for normally distributed continuous variables, a Mann–Whitney test for skewed variables, and a Chi-square test for nominal variables. The Kaplan–Meier analysis was used to estimate the freedom of arrhythmia recurrences during follow-up period. The differences between low (0–1), intermediate (2–3) and high risk (4–5) accordingly to APPLE and MB-LATER scores were compared using log-rank analysis.

Cox regression analysis was used to identify factors associated with arrhythmia recurrences >3 months during the whole follow-up. Multivariable analysis, which included variables with a p-value < 0.1 found on univariable analysis, was performed to identify independent predictors of recurrences.

Receiver operating characteristic (ROC) curves were generated for graphical illustration of APPLE, MB-LATER and modified scores’ performance in predicting rhythm outcome, with the area under the curve (AUC) being equivalent to the c-index for determining the predictive value for a score.

A p-value < 0.05 was considered as statistically significant. Statistical analyses were performed with IBM SPSS Statistics for Windows Version 25 (IBM Corp, Armonk, NY, USA).

## Results

The study population included 879 patients. Baseline characteristics are presented in Table 1. Median age was 61 (IQR 54–68) years and 560 (64%) were males. The median observed follow-up time was 37 months with 95% CI [35;39]. ERAF and LRAF occurred in 45% and 64%, respectively. There were 89 (10%) and 98 (11%) patients receiving antiarrhythmic drugs at the discharge because of complicated ablation and/or operator’s decision and at 3 months follow-up, respectively. Among them 69 (78%) and 81 (83%), respectively, had arrhythmia recurrences >3 months period (both p < 0.05).

**Table 1:** Baseline characteristics of the study population.

n (%) or median (IQR)	Total population n = 879	No recurrences n = 239	Only ERAF n = 78	Only LRAF n = 248	ERAF and LRAF n = 314	*p*-value
Age, years	61 (54–68)	60 (53–67)	60 (54–69)	62 (53–69)	63 (56–69)	0.034
Males	560 (64)	152 (64)	51 (65)	167 (67)	187 (60)	0.688
Persistent AF	339 (39)	58 (24)	27 (35)	97 (39)	157 (50)	<0.001
BMI, kg/m^2^	28 (25–31)	28 (25–30)	28 (25–30)	28 (26–31)	28 (26–31)	0.438
BMI ≥30 kg/m²	290 (33)	70 (29)	20 (26)	89 (36)	111 (35)	0.056
Hypertension	649 (74)	171 (72)	57 (73)	178 (72)	243 (77)	0.701
Diabetes mellitus	154 (18)	34 (14)	10 (13)	44 (18)	66 (21)	0.129
Coronary artery disease	128 (15)	31 (13)	6 (8)	42 (17)	49 (16)	0.122
Chronic herat failure	61 (7)	6 (3)	4 (5)	18 (7)	33 (11)	0.008
Peripheral artery disease	87 (10)	18 (8)	8 (10)	30 (12)	31 (10)	0.110
eGFR, ml/min/1.73 m^2^	96 (79–118)	99 (83–119)	94 (76–121)	99 (77–120)	95 (79–114)	0.634
eGFR <60 ml/min/1.73 m^2^	64 (7)	9 (4)	4 (5)	24 (10)	27 (9)	0.006
LA diameter (AP), mm	42 (39–46)	41 (37–45)	42 (39–46)	43 (39–47)	43 (40–48)	<0.001
EF, %	60 (55–65)	61 (57–65)	62 (56–66)	60 (55–65)	60 (53–65)	0.001
Bundle brunch block	60 (7)	14 (6)	2 (3)	19 (8)	25 (8)	0.089
CHA_2_DS_2_-VASc score	2 (1–3)	2 (1–3)	2 (1–3)	2 (1–3)	2 (1–3)	0.004
APPLE score	1 (1–2)	1 (0–2)	1 (1–2)	2 (1–2)	2 (1–2)	<0.001
MB-LATER score	2 (1–2)	1 (0–2)	2 (1–3)	1 (1–2)	2 (2–3)	<0.001

Abbreviations: IQR – interquartile range, AF – atrial fibrillation, BMI – body mass index, eGFR – estimated glomerular filtration rate, LA – left atrial, AP – antero-posterior, EF – ejection fraction.

There were 239 (27%) without any recurrences during follow-up, 78 (9%) with ERAF only, 248 (28%) with LRAF only, and 314 (36%) with both. Patients with LRAF were significantly older, had more often persistent AF, chronic heart failure, larger LA diameter and higher CHA_2_DS_2_-VASc, APPLE and MB-LATER scores than patients with sinus rhythm during follow-up (Table 1).

### Prediction of arrhythmia recurrences using clinical variables

On univariable Cox regression analysis, age, persistent AF type, diabetes mellitus, coronary artery disease, eGFR <60 ml/min/1.73 m², LA diameter, and ERAF were significant time-dependent predictors of arrhythmia recurrences >3 months after ablation (Table 2).

**Table 2:** Prediction of arrhythmia recurrences >3 months using clinical variables.

Variables	Univariable analysis		Multivariable analysis	
	HR (95% CI)	*p*-value	HR (95% CI)	*p*-value
Age, years	1.015 (1.006–1.024)	0.001	1.002 (0.992–1.012)	0.656
Females	1.178 (0.992–1.400)	0.062	1.329 (1.093–1.616)	0.004
Persistent AF	1.608 (1.361–1.901)	<0.001	1.425 (1.192–1.703)	<0.001
BMI ≥30, kg/m^2^	1.183 (0.995–1.407)	0.057	1.050 (0.867–1.271)	0.618
Hypertension	1.148 (0.947–1.391)	0.160		
Diabetes mellitus	1.304 (1.058–1.607)	0.013	1.056 (0.847–1.315)	0.629
Coronary artery disease	1.469 (1.172–1.842)	0.001	1.454 (1.148–1.842)	0.002
Peripheral artery disease	1.266 (0.970–1.652)	0.083	1.157 (0.877–1.527)	0.302
eGFR <60 ml/min/1.73 m^2^	1.490 (1.117–1.988)	0.007	1.380 (1.005–1.894)	0.046
LA diameter, mm	1.034 (1.020–1.048)	<0.001	1.022 (1.006–1.038)	0.006
EF, %	1.000 (0.999–1.001)	0.924		
Bundle brunch block	1.251 (0.996–1.572)	0.054	1.230 (0.973–1.554)	0.084
ERAF	2.270 (1.920–2.684)	<0.001	2.081 (1.749–2.476)	<0.001

On multivariable analysis, female sex (HR 1.329, 95% CI 1.093–1.616, p < 0.001), persistent AF type (HR 1.425, 95% CI 1.192–1.703, p < 0.001), coronary artery disease (HR 1.454, 95% CI 1.148–1.842, p = 0.002), eGFR <60 ml/min/1.73 m² (HR 1.380, 95% CI 1.005–1.894, p = 0.046), LA diameter (HR 1.022, 95% CI 1.006–1.038, p = 0.006), and ERAF (HR 2.081, 95% CI 1.749–2.476, p < 0.001) remained significant predictors for LRAF, while bundle brunch block did not reach significance (HR 1.2, 95%CI 0.973–1.554, p = 0.084).

**Figure 1:**
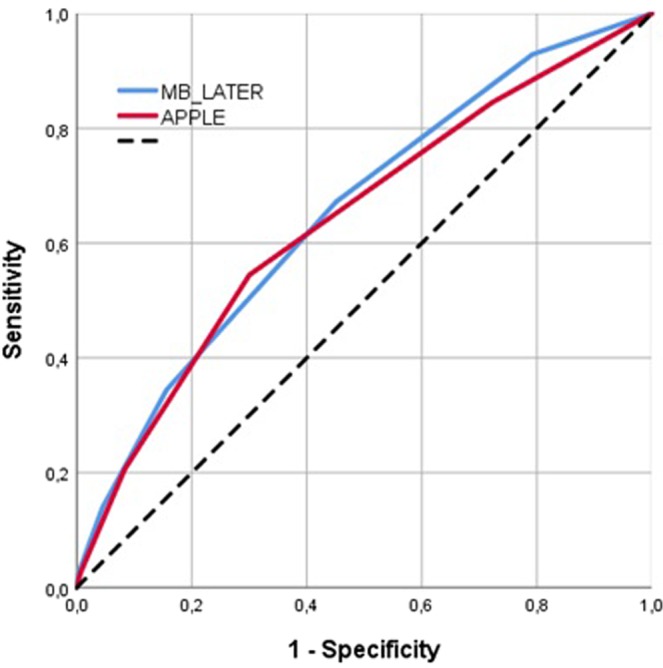
ROC curves analysis for prediction of arrhythmia recurrences >3 months.

The APPLE (HR 1.385, 95% CI 1.276–1.505, p < 0.001) and MB-LATER (HR 1.326, 95% CI 1.239–1.419, p < 0.001) scores significantly predicted arrhythmia recurrences >3 months after ablation (Table 3). On ROC analyses, both scores demonstrated moderate predictive value for both scores: APPLE AUC 0.640 (95% CI 0.602–0.677, p < 0.001) and MB-LATER AUC 0.654 (95% CI 0.616–0.691, p < 0.001, Table 3, Fig. 1).

**Table 3:** Prediction of arrhythmia recurrences >3 months using scores.

Scores	HR (95% CI)		AUC (95% CI)	p-value
APPLE	1.385 (1.276–1.505)	<0.001	0.640 (0.602–0.677)	<0.001
MB-LATER	1.326 (1.239–1.419)	<0.001	0.654 (0.616–0.691)	<0.001
CHA_2_DS_2_-VASc	1.159 (1.097–1.225)	<0.001	0.572 (0.534–0.611)	<0.001

### Kaplan-Meier analysis

**Figure 2:**
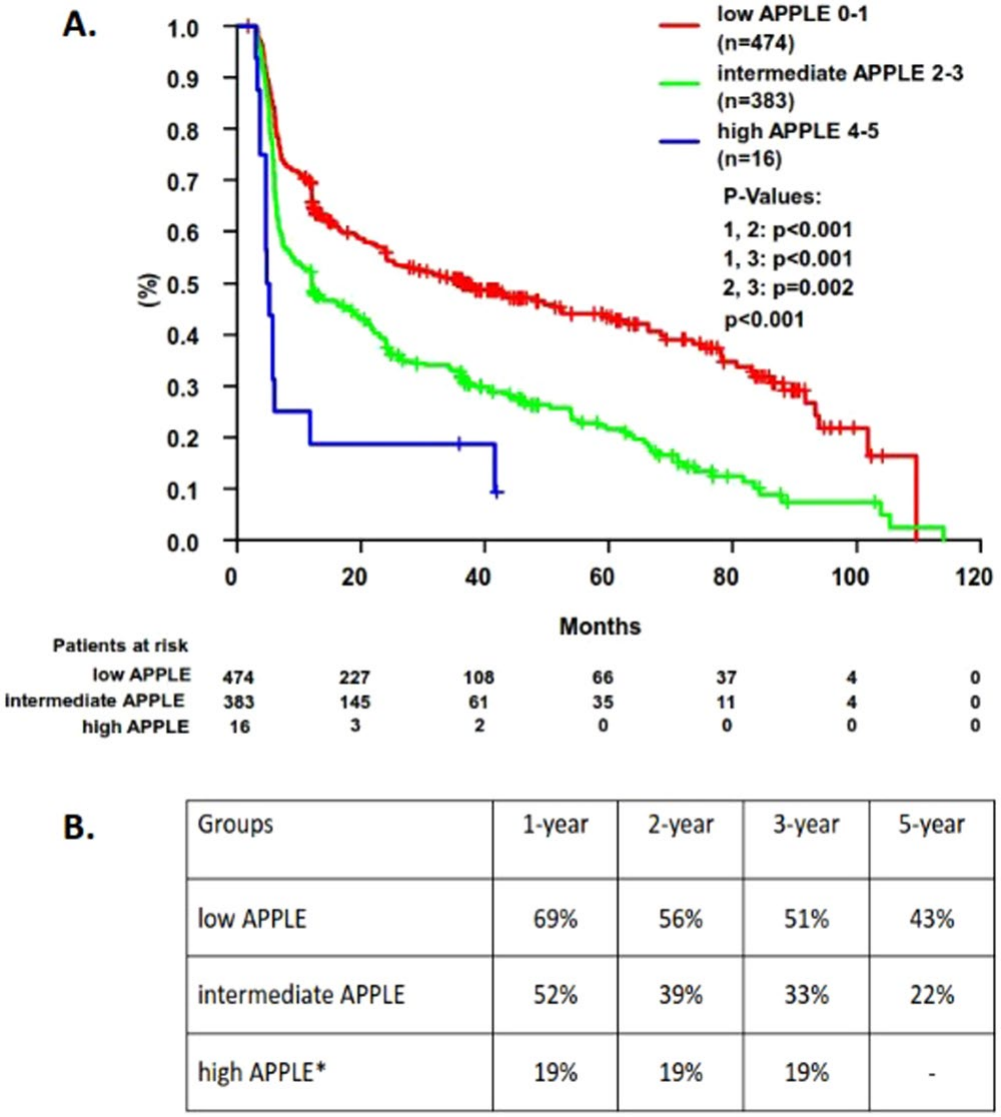
(**A**) Prediction of arrhythmia recurrences >3 months using APPLE score low, intermediate and high strata during follow-up (**B**). Probability for AF freedom at 1-, 2-, 3-, and 5-years accordingly to the APPLE score strata. *small sample size (<5 patients).

**Figure 3:**
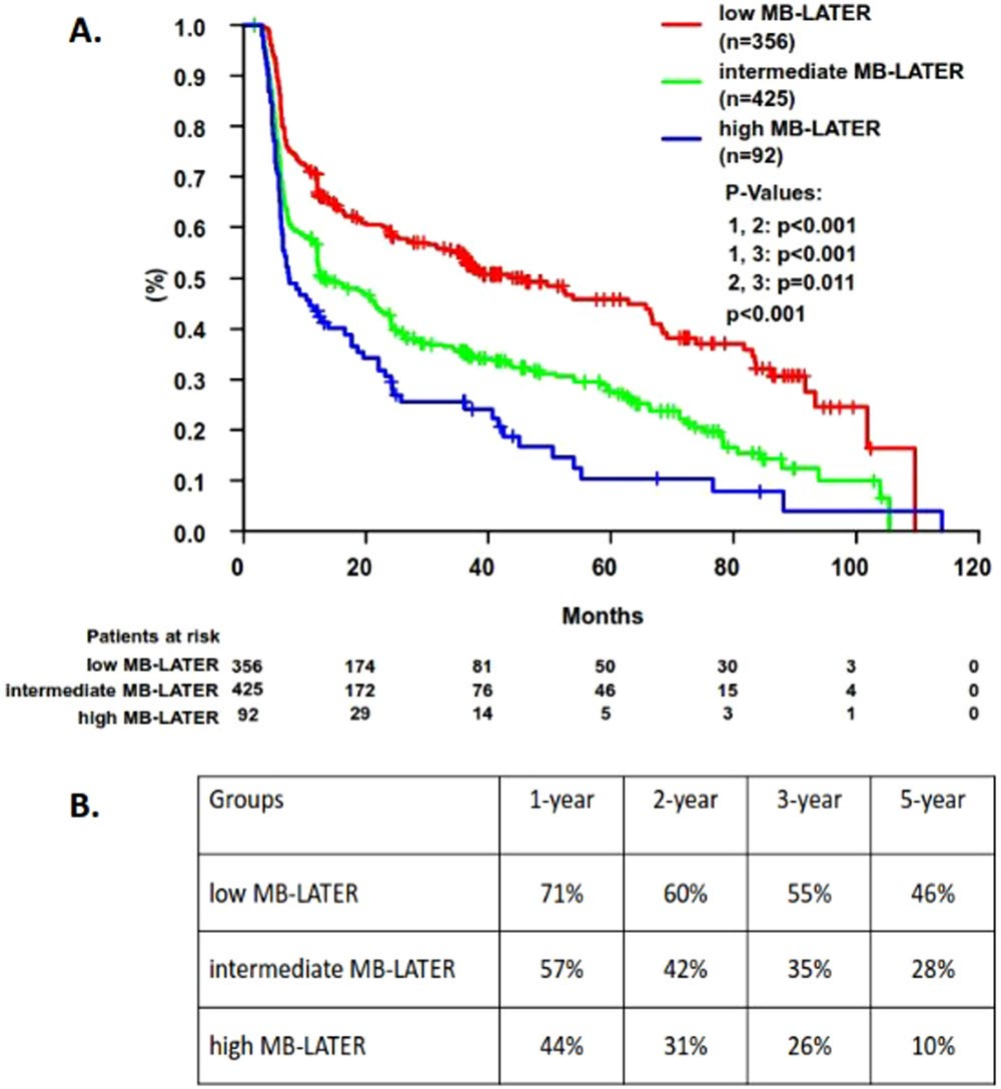
(**A**) Prediction of arrhythmia recurrences >3 months using MB-LATER score low, intermediate and high strata during follow-up (**B**). Probability for AF freedom at 1-, 2-, 3-, and 5-years accordingly to the MB-LATER score strata.

Using Kaplan-Meier curves, we analyzed the risk of late arrhythmia recurrences in patients accordingly to the low (0–1), intermediate (2–3) and high risk (4–5) accordingly to the APPLE and MB-LATER scores during follow-up. As expected, patients with lower risk (APPLE 0–1 and MB-LATER 0–1) had significantly better rhythm outcomes than patients with intermediate (APPLE and MB-LATER 2–3) or higher risk (APPLE and MB-LATER 4–5; log-rank <0.001, Figs 2–3).

There were 69%, 56%, 51%, and 43% probability for the arrhythmia freedom at 1-, 2-, 3- and 5-years for the low APPLE strata, while for the patients with intermediate APPLE score – 52%, 39%, 33%, and 22% for the same follow-up period (Fig. 2). Similarly, the arrhythmia freedom at 1-, 2-, 3- and 5-years for the low MB-LATER strata was 71%, 60%, 55%, and 46%, while patients with intermediate MB-LATER score remained at sinus rhythm at 57%, 42%, 35%, and 28% (Fig. 3).

## Discussion

To the best of our knowledge, the current study is the first one analyzing the time-dependent prognostic ability of APPLE and MB-LATER scores for the prediction of arrhythmia recurrences after first radiofrequency catheter ablation during long term follow-up. First, we found that both scores were associated with rhythm outcomes demonstrating moderate predictive ability. Furthermore, the rate of arrhythmia-free survival after catheter ablation depends on the follow-up length and the scores strata. Finally, among clinical variables female sex, coronary artery disease, renal dysfunction, LA diameter, and ERAF were important factors associated with LRAF in a time-dependent manner.

### Prediction of arrhythmia recurrences

Despite advanced mapping, ablation catheters and techniques, arrhythmia recurrences still remain a common issue after catheter ablation. Therefore, there is a considerable clinical interest to predict the risk of recurrences occurrence already before procedure. Despite the availability of multiple rhythm prediction scores, the prediction of arrhythmia recurrences remains challenging and not in a wide usage in a daily clinical routine.

During the recent years, several studies demonstrated the impact of diverse clinical scores on recurrence prediction. First, the thromboembolic risk predicting CHADS_2_ and CHA_2_DS_2_-VASc scores showed relatively modest prediction for arrhythmia recurrences^7,8^. Later, several specific rhythm outcomes prediction scores as ALARMEC, BASE-AF2, CAAP-AF, ATLAS and LAGO had been introduced^6,9–12^. However, the results of these studies are partly difficult to interpret because of some non-standardized definitions, relatively small study populations and short follow-up period. However, the direct comparison of the scores is difficult because of different cohort sizes and different AF ablation protocols. Also, only few scores had such a quality criterion as the external validation. In AF patients undergoing radiofrequency ablation only APPLE and MB-LATER scores were validated in several external cohorts^13^.

The APPLE and MB-LATER scores were both introduced as rhythm outcomes predicting scores. While the APPLE score predicted arrhythmia recurrences within one year in patients undergoing first and repeat ablation^14,17^, the MB-LATER score had been developed for the prediction of arrhythmia recurrences over 12 months in patients with arrhythmia-free survival within first year^15^.

In current study, we found that several clinical factors, including persistent AF type, LA diameter and ERAF were significantly associated with arrhythmia recurrences during long-term follow-up. These results are in line with our previous study and recent meta-analysis^18,19^ Importantly, persistent AF type was included into both APPLE and MB-LATER scores and remained significant time-dependent predictor for the arrhythmia recurrences. This could be explained by higher prevalence of low voltage areas in patients with persistent AF type. However, this can be only speculated as a decade ago an electro-anatomical mapping was not routinely performed in the study cohort.

### ERAF as a time-dependent predictor of arrhythmia recurrences

It had been already demonstrated that patients with ERAF are more likely to suffer from long-term arrhythmia recurrences^20^. Nevertheless, in more than half of patients with ERAF a delayed cure during subsequent follow-up is possible^21^. Hence, a blanking period of 3 months is considered as meaningful to avoid unnecessary repeat procedures. Recent studies found that up to 67% of patients with ERAF suffered LRAF during longer follow-up^22^, and a recurrence free-rate in persistent AF after single procedure was only in 28%^23^. These findings are in line with our current results. We found significant differences in clinical profiles between patients without recurrences, with ERAF or LRAF only, and with both recurrences. Patients suffering LRAF and ERAF/LRAF were significantly older, had more often persistent AF, larger LA diameter and higher APPLE and MB-LATER scores than patients without recurrences at all or only with ERAF. Furthermore, we demonstrate that the arrhythmia-free survival changed significantly during follow-up period reaching 43% and 22% for low and intermediate APPLE or 46% and 28% for low and intermediate MB-LATER strata at 5-years milestone.

In our recent study, the ERAF demonstrated the best association with LRAF in a contemporary AF ablation cohort and in the retrospective validation subgroup from The Leipzig Heart Center AF Ablation Registry^16^. Also, we found that patients with ERAF had almost 4-fold risk for LRAF occurrence during follow-up^19^. This had been also confirmed by a meta-analysis^18^ and in our current study. However, ERAF is not available at baseline, and MB-LATER cannot be calculated before catheter ablation – in contrast to the APPLE score. Nevertheless, a relatively moderate predictive value of both scores in ROC analysis indicates that a robust rhythm outcome predicting score still remains a clinical unmet need. The role of other variables – especially imaging, biomarkers, electro-anatomical features – should be analysed building a novel or improving previous rhythm outcome scores.

### Study limitations

This study is limited by its observational, retrospective design in a single-center cohort. First, the majority of the patients were not monitored by loop recorders; asymptomatic episodes of silent AF could have been missed. Second, there were only patients undergoing first radiofrequency AF catheter ablation. The results could differ in patients with repeated procedures or with ablation using other sources. Third, the electro-anatomical mapping data were not routinely performed in the whole study cohort. Furthermore, the data of obstructive sleep apnoea syndrome, thyroid disease and sick sinus syndrome, which may have an impact on AF progression and occurrence of arrhythmia recurrences after catheter ablation, were not available in this study. Also, because of very low patient numbers with high risk accordingly to the APPLE score (e.g. APPLE 4–5) including only 16 patients, a robust statistical analysis for this subgroup was not possible. The prediction of arrhythmia recurrences for these patients should be evaluated in larger prospective studies including larger numbers of such sub-cohort. Finally, whether APPLE and MB-LATER score have enough strength to be considered for explaining the patients the expected outcome of the procedure, and therefore to balance advantages and disadvantages when considering a patient for AF ablation, could be answered only in controlled randomized trials addressing this issue.

## Conclusions

APPLE and MB-LATER scores were useful for the time-dependent prediction of arrhythmia recurrences in patients undergoing first AF catheter ablation. The rate of arrhythmia-free survival after catheter ablation depends on the follow-up length and the scores strata.

## Data Availability

All data generated or analyzed during this study are included in this published article.
